# Ultrasound assessment of lower limb muscle mass in response to resistance training in COPD

**DOI:** 10.1186/1465-9921-13-119

**Published:** 2012-12-28

**Authors:** Manoj K Menon, Linzy Houchen, Samantha Harrison, Sally J Singh, Michael D Morgan, Michael C Steiner

**Affiliations:** 1 Department of Respiratory Medicine, University Hospitals of Leicester NHS Trust, Glenfield Hospital, Groby Road, Leicester, LE3 9QP, UK; 2 Physiological Interventions Research Group, Coventry University, Coventry, UK

**Keywords:** COPD, Quadriceps, Rectus femoris, Ultrasound, Resistance training

## Abstract

**Background:**

Quantifying the improvements in lower limb or quadriceps muscle mass following resistance training (RT), is an important outcome measure in COPD. Ultrasound is a portable, radiation free imaging technique that can measure the size of superficial muscles belonging to the quadriceps group such as the rectus femoris, but has not been previously used in COPD patients following RT. We compared the responsiveness of ultrasound derived measures of quadriceps mass against dual energy x-ray absorptiometry (DEXA), in patients with COPD and healthy controls following a programme of high intensity knee extensor RT.

**Methods:**

Portable ultrasound was used to assess the size of the dominant quadriceps in 45 COPD patients and 19 healthy controls-before, during, and after 8 weeks of bilateral high intensity isokinetic knee extensor RT. Scanning was performed at the mid-thigh region, and 2 indices of quadriceps mass were measured-rectus femoris cross-sectional area (RF_csa_) and quadriceps muscle thickness (Q_t_). Thigh lean mass (T_dexa_) was determined by DEXA.

**Results:**

Training resulted in a significant increase in T_dexa_, RF_csa_ and Q_t_ in COPD patients [5.7%, 21.8%, 12.1% respectively] and healthy controls [5.4%, 19.5%, 10.9 respectively]. The effect size for the changes in RF_csa_ (COPD= 0.77; Healthy=0.83) and Q_t_ (COPD=0.36; Healthy=0.78) were greater than the changes in T_dexa_ (COPD=0.19; Healthy=0.26) following RT.

**Conclusions:**

Serial ultrasound measurements of the quadriceps can detect changes in muscle mass in response to RT in COPD. The technique has good reproducibility, and may be more sensitive to changes in muscle mass when compared to DEXA.

**Trial registration:**

http://www.controlled-trials.com (Identifier: ISRCTN22764439)

## Introduction

Reduced lower limb skeletal muscle mass and strength is an important systemic feature of Chronic Obstructive Pulmonary Disease (COPD) which has a significant impact on mortality, morbidity and healthcare utilisation [1-4]. Improvements in muscle mass can be achieved by lower limb resistance training. These improvements are restricted to the muscle group that is trained. Hence, a reliable, safe measurement of lower limb or quadriceps muscle mass which can detect the response to an intervention such as exercise training is needed.

Muscle mass can be measured using a variety of imaging techniques such as computed tomography (CT), magnetic resonance imaging (MRI) and dual energy x-ray absorptiometry (DEXA). In COPD, previous studies have suggested that CT, MRI and DEXA can all detect changes in lower limb muscle mass following training [5-7]. However, the equipment required for these measurements is bulky and expensive, specific expertise may be required to interpret the images, and in the case of CT and DEXA, subjects are exposed to ionizing radiation. Thus the utility of these imaging modalities as an outcome measure, where repeat testing is required, is limited. Ultrasound is an imaging technique that can determine thickness and cross-sectional areas of superficial muscles such as the rectus femoris muscle. It has the advantage of being portable, and involves no ionizing radiation. A number of studies have confirmed the reliability of this technique for measuring the size of the quadriceps muscle in health [8-10], with limited data in COPD [11]. Similarly, ultrasound has been previously shown to detect changes in quadriceps size in response to training interventions in healthy populations [12-14]. However, no comparable data exists in COPD, where muscle mass is lower and training intensities reduced. Seymour et al. observed good correlation between ultrasound measurements of rectus femoris cross-sectional area and CT in a cohort of patients with COPD but the responsiveness of this measure to an intervention that increases muscle mass has not been assessed [11].

In this study, we compared the responsiveness of ultrasound and DEXA assessments of lower limb muscle mass in response to high intensity knee extensor RT, in patients with COPD and a similar aged healthy control group. We hypothesized that ultrasound derived surrogates of muscle mass, namely rectus femoris cross-sectional area and quadriceps thickness, would be sensitive to changes in response to RT. In addition we assessed the inter-operator and inter-occasion reproducibility of the ultrasound technique at baseline, and compared its performance to DEXA as a means of measuring thigh muscle size.

## Methods

### Study subjects

45 patients with COPD and 19 age-matched controls were included in this study. They were all participating in a larger investigation of the mechanisms of adaptation to RT. Patients were recruited from outpatient clinics at Glenfield Hospital (Leicester, UK), and from those referred for pulmonary rehabilitation. Age-matched healthy controls were recruited from local advertisement. None of the subjects had been taking part in any regular exercise programs, and COPD patients who underwent pulmonary rehabilitation in the last 12 months were excluded. Other exclusion criteria included: maintenance oral corticosteroid or anticoagulant therapy, long-term oxygen therapy, diabetes or any other co morbid conditions that would prevent exercise training. The study was approved by the Leicestershire and Rutland Research Ethics committee (Ref: 06/Q2501/138), and all participants provided written informed consent.

### Lung function

Spirometry was measured in the seated position (Model R; Vitalograph, Buckingham, UK) according to standards set out by the European Respiratory Society [15].

### Resistance training protocol

Participants underwent 8 weeks of bilateral, knee extensor, high-intensity isokinetic RT on an isokinetic dynamometer (Cybex II Norm, CSMi, Stoughton, MA, USA). Training was fully supervised, and consisted of three half-hour sessions per week. Subjects performed 5 sets of 30 maximal knee extensions at a pre-set angular velocity (PAV) of 180°/second. The contractions were isokinetic and concentric, and each set was separated by a minutes rest. Additionally, subjects also received one-minute continuous passive movement (flexion/extension) before and after each training session to act as a warm up/cool-down. This training protocol was chosen based upon a previous study showing that the training could produce significant increases in lower limb mass following immobilisation in healthy subjects [16]. The basic measurements recorded were the peak torque in Newton-metres (Nm) and total work done in Joules (J) for each of the five sets.

### Measurements pre and post training

(a) Thigh Muscle Mass

#### DEXA

Total body lean (fat free) mass was measured by DEXA (Lunar Prodigy Advance, GE Healthcare, UK). This provides a 3-compartment model of body composition, subdividing the body into fat mass, bone-free lean mass and bone mineral mass. Using the software provided by the manufacturer, thigh lean mass (T_dexa_) was measured from the area delineated by the ischeal tuberosity superiorly, and knee joint line inferiorly [17]. The fat free mass index (FFMI) was calculated from the total body fat free mass normalised for height. Patients were deemed to be muscle wasted if the FFMI < 16 kg/m^2^ in men or <15 kg/m^2^ in women [18].

#### Ultrasound

**Figure 1:**
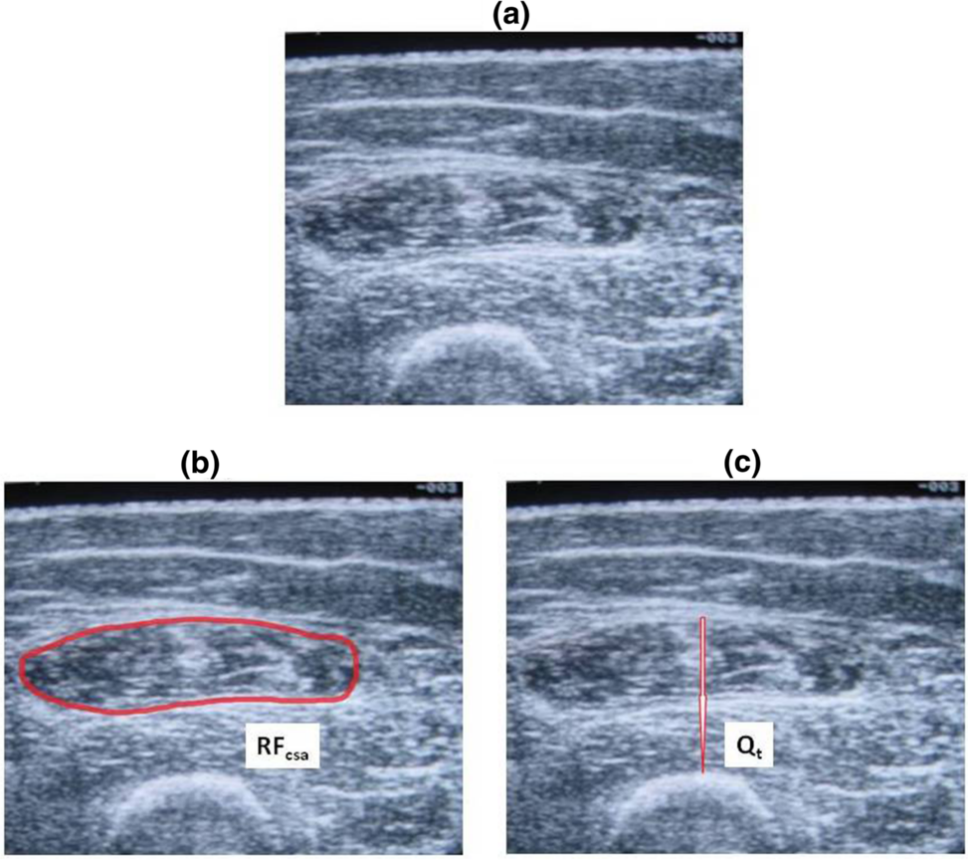
**Ultrasound image of the quadriceps.** Sample scan of a study participant showing (**a**) Image at mid-thigh region, (**b**) RF_csa_, and (**c**) Q_t_.

Portable ultrasound (Hitachi EUB-425, Hitachi Medical Systems, UK) was used to measure the size of the dominant quadriceps muscle similar to the method of Bemben et al. [8]. Scanning was performed in the supine position with a rolled-up towel placed in the popliteal fossa to relax the upper thigh. The scanning site was identified as the mid-point of the distance from the greater trochanter to the knee joint line as previously described [19]. A 7.5 MHz linear array transducer was placed perpendicular to the long axis of the thigh to obtain a frozen real-time cross-sectional image of the rectus femoris muscle. Using the built-in callipers, two indices of quadriceps size were measured – rectus femoris cross-sectional area-RF_csa_, and quadriceps thickness-Q_t_. The inner outline of the rectus femoris was manually traced to calculate RF_csa_ in mm^2^, while Q_t_ was measured in mm as the vertical distance from the superficial fat-muscle interface to the underlying femur (Figure 1). The average of three consecutive measurements (within 10% of one another) was taken as the true value. Care was taken to ensure that adequate contact gel was used and minimal pressure applied on the transducer so as to minimise distortion of underlying tissues. Scans were performed at baseline, and after weeks 4 and 8 of training. In a subset of participants, the baseline scans were repeated by 2 additional operators (LH and SH) in order to assess inter-operator reproducibility of the method. Each operator was blind to the other’s scans. In addition, a repeat baseline scan was performed by MM on a separate visit prior to the start of exercise training to determine inter-occasion reproducibility of the ultrasound technique. None of the operators had any previous ultrasound experience, but following a brief familiarisation period, competency was gained in performing the scans independently.

(b) Quadriceps Strength

Quadriceps strength was measured on the cybex. After a prior familiarisation visit, quadriceps isometric maximum voluntary contraction (QMVC) of the dominant leg was measured during a maximal static contraction with the knee at 70°. QMVC was measured in Newton-metres (Nm), and the best of 6 strength measurements was taken as the true value.

#### Statistics

Statistical analysis was performed using GraphPad Prism Version 5.01 for Windows (GraphPad Software Inc, California, USA) and SPSS 18.0 for Windows (SPSS Inc, Chicago, USA). Parametric data were expressed as means (± SD) and non-parametric data were described as medians (interquartile range, ±IQR). Spearman’s correlation was used to describe the relationship between changes in RF_csa_, Q_t_ and QMVC. The reproducibility of ultrasound measurements was determined by calculating the mean differences and the intraclass correlation coefficients for repeated measurements. The effect size for changes in outcome measures after resistance training was calculated by dividing the mean difference by the standard deviation of the pre-training measurement. By calculating effect sizes, the magnitude of any changes can be judged according to the following criteria-small: 0.2 to 0.5; moderate: 0.5 to 0.8; large: > 0.8 [20]. All statistical tests were two-tailed and the threshold of statistical significance was a p value < 0.05.

## Results

### Baseline

**Figure 2:**
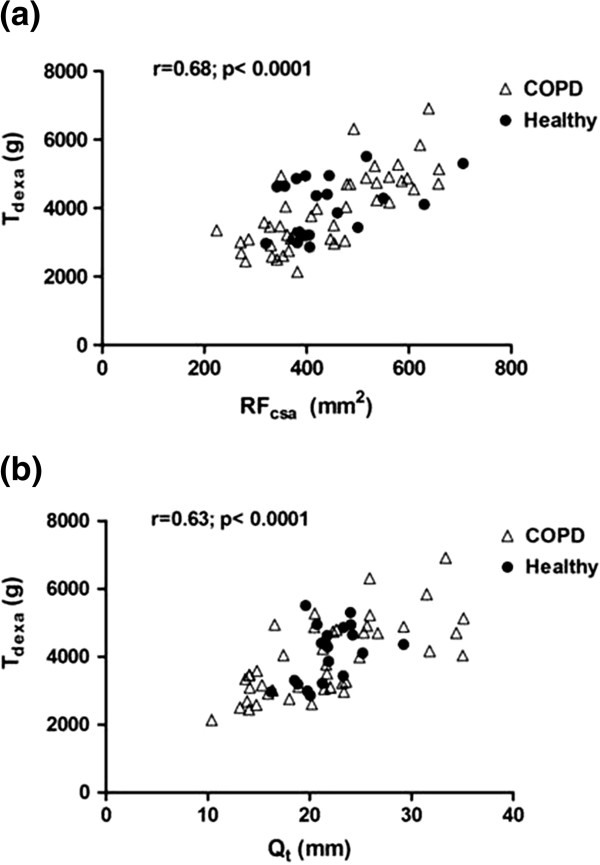
**Baseline relationships between ultrasound and DEXA measured indices of quadriceps size (a) T**_**dexa VS. **_**RF**_**csa **_**and (b) T**_**dexa **_**vs. Q**_**t**_**.**

**Figure 3:**
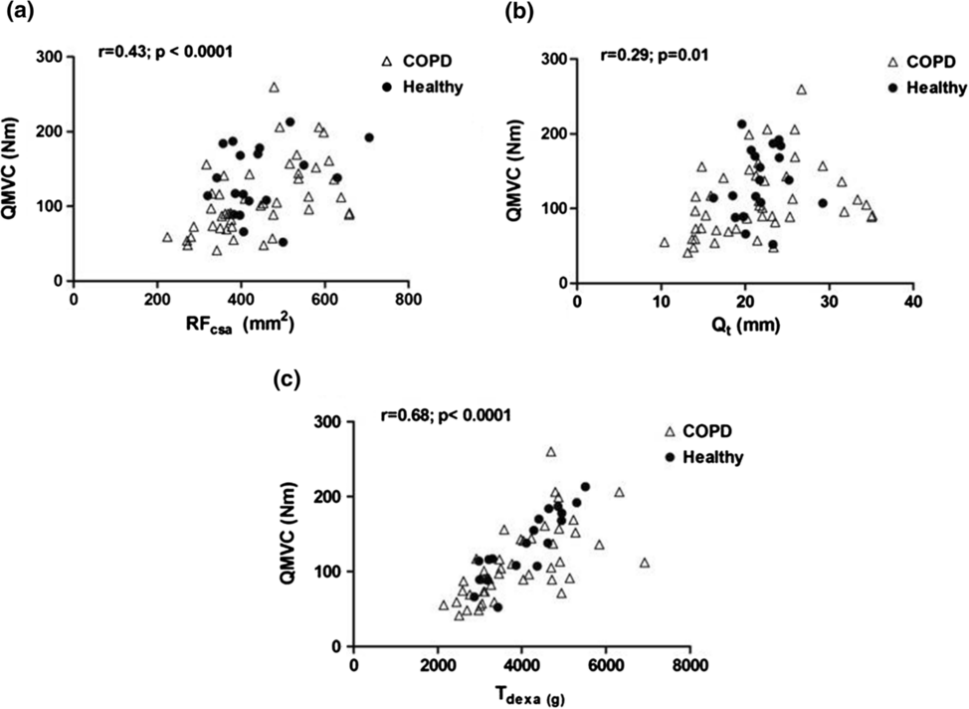
**Baseline relationships between quadriceps strength and ultrasound and DEXA measured indices of quadriceps mass (a) QMVC vs. RF**_**csa**_**, (b) QMVC vs. Q**_**t**_**, and (c) QMVC vs. T**_**dexa**_**.**

Baseline subject characteristics are shown in Table 1. QMVC was significantly lower in patients compared with controls, but ultrasound and DEXA indices of thigh muscle mass did not differ between the groups at baseline. There were 10 muscle wasted COPD patients. For the group as a whole, a significant linear relationship was observed between ultrasound and DEXA measured indices of quadriceps size [RF_csa_ vs. T_dexa_: r=0.68, p<0.0001; Q_t_ vs. T_dexa_: r=0.63, p<0.0001 – Figure 2a and b]. Both RF_csa_ and Q_t_ were significantly related to QMVC with the groups combined, [RF_csa_: r=0.43, p< 0.0001; Q_t_: r = 0.29, p= 0.01 – Figure 3a and b] and when taking into account only COPD patients [RF_csa_: r=0.50, p= 0.0005; Q_t_: r=0.33, p=0.02]. A significant but stronger correlation was observed between QMVC and T_dexa_ [r=0.68; p <0.0001 – Figure 3c].

**Table 1:** Baseline characteristics

	**Healthy (n=19)**	**COPD (n=45)**
Age	66.2 (5.0)	68.2 (8.2)
Gender (M:F)	8:11	27:18
Smoking (pack-years)	13.0 (22.4)	45.8 (30.5)***
FEV_1_ (L)	2.5 (0.6)	1.1 (0.4)***
FEV_1_ (% predicted)	106.6 (22.0)	47.3 (18.9)***
BMI (kg/m^2^)	26.9 (2.8)	26.4 (5.3)
FFMI (kg/m^2^)	17.3 (1.7)	17.5 (2.8)
QMVC (Nm)	135.7 (45.7)	109.5 (48.5)*
T_dexa_ (g)	4096.8 (849.3)	3908.5 (1104.1)
RF_csa_ (mm^2^)	444.1 (98.8)	439.7 (117.9)
Q_t_ (mm)	21.8 (2.9)	21.5 (6.4)

Data presented as Means (SD), except gender (absolute numbers).

* p<0.05, *** p<0.0001, COPD vs. Healthy.

**BMI**: Body mass index.

**FFMI**: Fat-free mass index.

**FEV**_**1**_: Forced expiratory volume in 1 second.

**QMVC**: Quadriceps maximum voluntary contraction.

**T**_**dexa**_: Thigh lean mass.

**RF**_**csa**_: Rectus femoris cross-sectional area measured by ultrasound.

**Q**_**t**_: Quadriceps muscle thickness measured by ultrasound.

### Post-training

**Figure 4:**
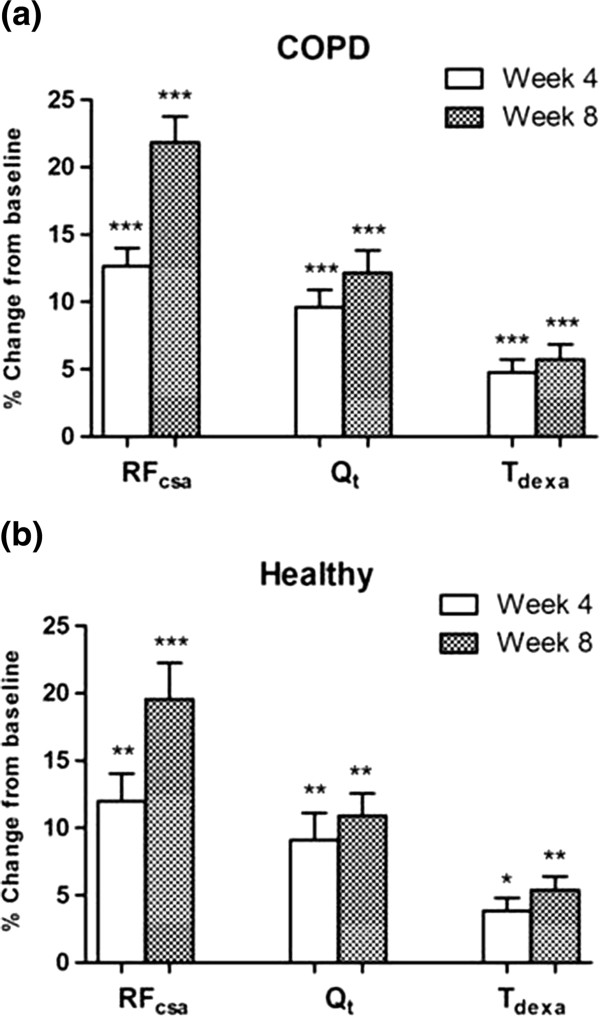
**Percentage change from baseline in ultrasound and DEXA measured indices of quadriceps mass in (a) COPD, and (b) Healthy.** Data presented as Means (SEM) **RF**_**csa**_: Rectus femoris cross-sectional area measured by ultrasound, **Q**_**t**_: Quadriceps muscle thickness measured by ultrasound, **T**_**dexa**_: Thigh lean mass measured by DEXA, Wilcoxon Signed rank test: *** p ≤ 0.0001; ** p < 0.001; * p < 0.01, significantly different from baseline.

**Figure 5:**
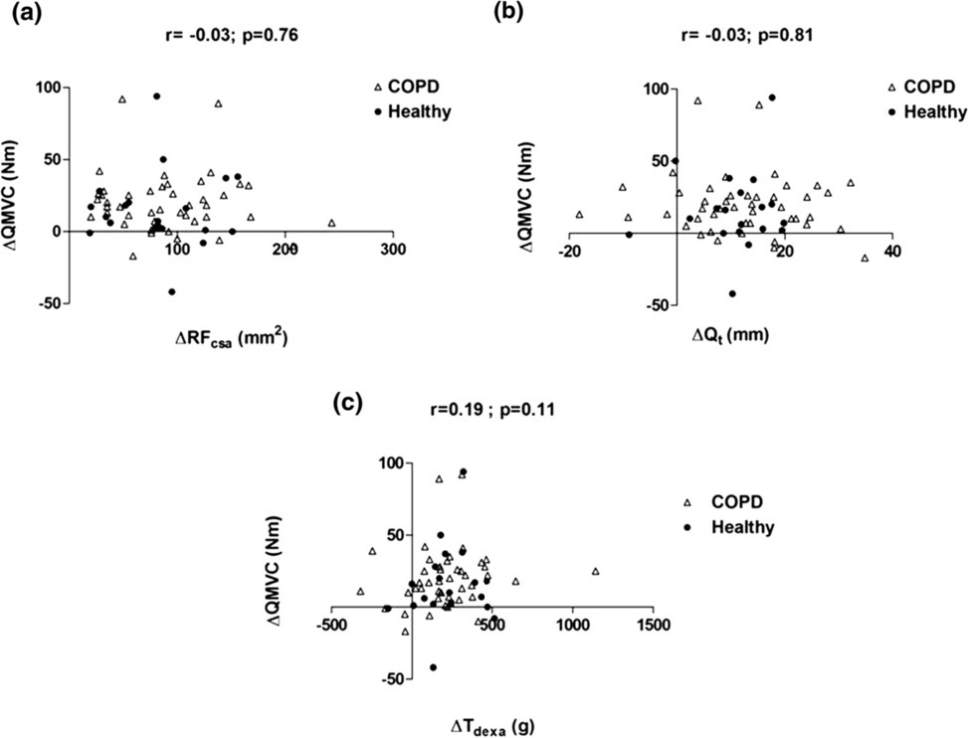
**Relationships between training induced changes in quadriceps strength (ΔQMVC) and (a) ΔRF**_**csa**_**, (b) ΔQ**_**t**_**, and (c) ΔT**_**dexa**_**.**

**Figure 6:**
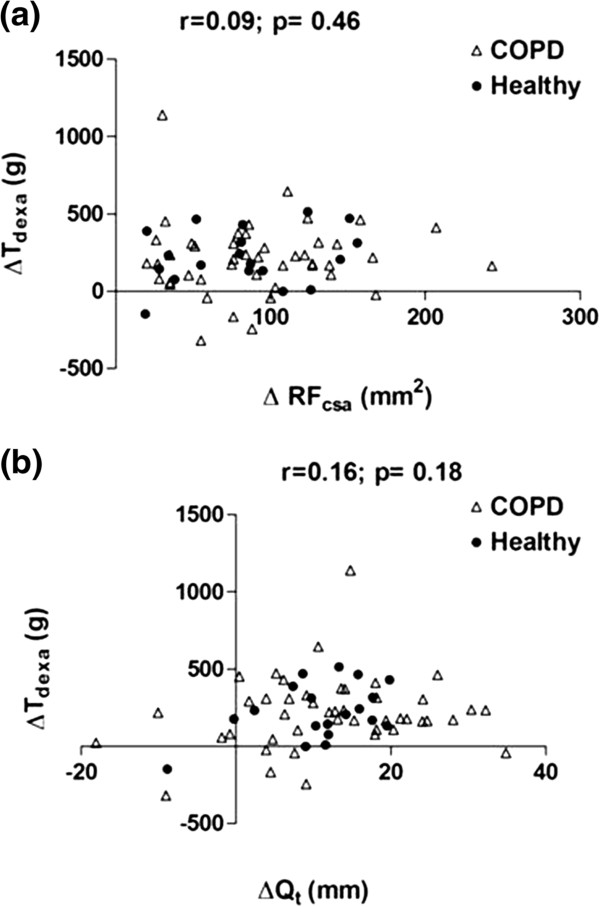
**The relationship between training-induced changes in ultrasound and DEXA indices of quadriceps mass (a) ΔT**_**dexa**_**vs. ΔRF**_**csa**_**, and (b) ΔT**_**dexa**_**vs. ΔQ**_**t**_**.**

The mean (SD) differences and effect sizes for measures of quadriceps mass and strength after training are shown in Table 2. An increase in quadriceps mass was detectable at the half-way stage (week 4) of the exercise training programme when measured by both ultrasound and DEXA. Eight weeks of RT resulted in a significant increase in T_dexa_, RF_csa_ and Q_t_ [COPD: 5.7%, 21.8%, 12.1% respectively; Healthy: 5.4%, 19.5%, 10.9 respectively] (Figure 4a and b). Similarly, QMVC significantly improved after training in both groups [Mean (SD) change-COPD: 19.5 (20.6) Nm, p<0.001; Healthy: 15.5 (27.5) Nm, p=0.02]. When compared to ultrasound, the post-training change in muscle mass measured by DEXA (T_dexa_) was more closely related to changes in muscle strength (r=0.19), although none of these relationships were statistically significant (Figure 5a, b and c). Similarly, training-induced changes in RF_csa_ and Q_t_ were not significantly correlated to changes in T_dexa._ (Figure 6a and b).

**Table 2:** Effect of training on quadriceps strength and mass

	**% change (SD)**	**Mean absolute change (SD)**	**95% CI**	**Effect size**	**p value**
**QMVC (Nm)**					
Healthy (n=19)	11.3 (19.6)	15.5 (27.5)	2.3, 28.8	0.34	0.024
COPD (n=45)	20.0 (20.4)	19.5 (20.6)	13.3, 25.7	0.40	0.000
**T**_**dexa**_**(g)**					
Healthy (n=19)	5.4 (4.2)	224.7 (178.9)	138.5, 311.0	0.26	0.000
COPD (n=45)	5.7 (7.6)	213.6 (235.0)	143.0, 284.2	0.19	0.000
**RF**_**csa**_**(mm**^**2**^**)**					
Healthy (n=19)	19.5 (11.6)	82.4 (44.3)	61.1, 103.8	0.83	0.000
COPD (n=45)	21.8 (12.7)	91.5 (50.3)	76.3, 106.6	0.77	0.000
**Q**_**t**_**(mm)**					
Healthy (n=19)	10.9 (7.1)	2.2 (1.5)	1.5, 3.0	0.78	0.000
COPD (n=45)	12.1 (11.2)	2.3 (2.2)	1.6, 3.0	0.36	0.000

Percentage and Mean absolute (SD) changes in outcome measures for healthy controls and COPD patients after 8 weeks of resistance training. 95% confidence intervals for the differences are quoted. p values are calculated using paired student’s t-tests.

### Muscle wasted patients

Ultrasound measured indices of quadriceps mass also improved significantly in the 10 muscle wasted COPD patients [RF_csa_: Mean (SD) from 356.6 (61.5) mm^2^ to 413.9 (76.1) mm^2^, p< 0.001; Q_t_: from 15.9 (4.0) mm to 18.1 (3.6) mm, p< 0.01], while nonsignificant improvements in T_dexa_ [from 2793.2 (419.3) g to 2983.9 (449.5) g, p=0.13] and QMVC [from 74.2 (34.6) Nm to 82.0 (30.8) Nm, p=0.08] were also noted.

### Reproducibility of ultrasound measurements

**Figure 7:**
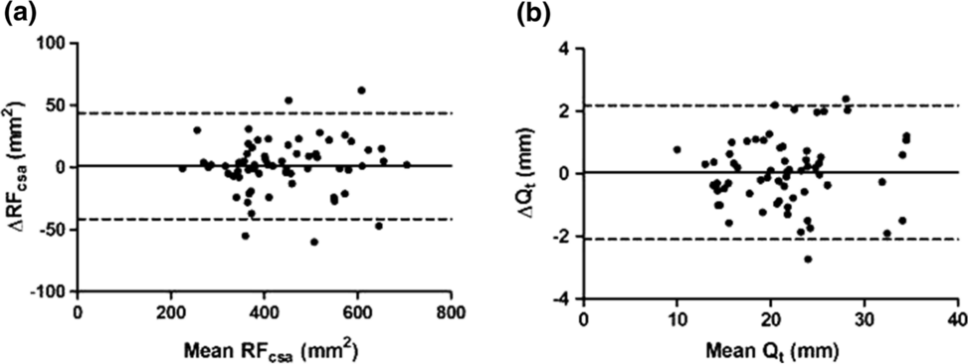
**Bland-Altman plots of the inter-occasion reproducibility of ultrasound measurements (a) RF**_**csa**_**and (b) Q**_**t**_**.**

**Figure 8:**
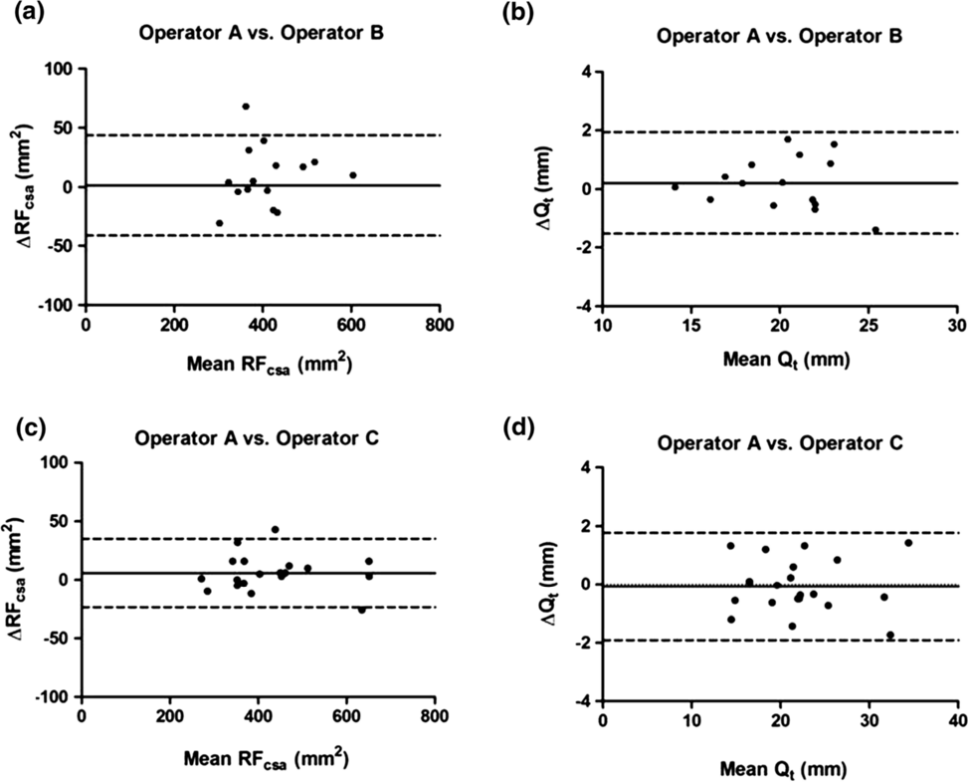
**Bland-Altman plots of the inter-operator reproducibility of ultrasound measurements (a) RF**_**csa**_**: Operator A vs. Operator B, (b) Q**_**t**_**: Operator A vs. Operator B, (c) RF**_**csa**_**: Operator A vs. Operator C, and (d) Q**_**t**_**: Operator A vs. Operator C.**

Table 3 summarises the results of reproducibility studies for the ultrasound measurements. Intraclass correlation coefficients and reliability indices between the interval scans for RF_csa_ and Q_t_ were 0.98, indicating good inter-occasion reproducibility of the technique for the same operator (Bland-Altman plots – Figure 7a and b). Inter-operator reproducibility was assessed between 2 pairs of operators [Operator A (MM) vs. Operator B (LH), and Operator A (MM) vs. Operator C (SH)] at baseline. Intraclass correlation coefficients and indices of reliability between operators were >0.95, indicating good inter-operator reproducibility for the ultrasound measurements (Bland-Altman plots Figure 8a, b, c and d).

**Table 3:** Reproducibility of ultrasound measurements

	Mean difference*		**SD**^**#**^		**p-value**		**ICC**	
	**RF**_**csa**_**(mm**^**2**^**)**	**Q**_**t**_**(mm)**	**RF**_**csa**_**(mm**^**2**^**)**	**Q**_**t**_**(mm)**	**RF**_**csa**_	**Q**_**t**_	**RF**_**csa**_	**Q**_**t**_
**Inter-occasion (n=64)**	1.18	0.04	21.69	1.09	0.66	0.75	0.98	0.98
**Inter-operator (A vs. B; n=15)**	8.73	0.20	25.39	0.88	0.20	0.38	0.95	0.95
**Inter-operator (A vs. C; n=20)**	5.90	0.07	15.01	0.93	0.09	0.74	0.99	0.98

*: Difference between measurements taken on 2 separate occasions at baseline (Test-Retest).

^**#**^: of mean difference.

Significance was tested using paired t tests.

ICC: Intraclass correlation coefficient.

## Discussion

We have shown that indices of quadriceps mass measured by ultrasound are sensitive to change in response to RT in COPD patients (including subjects with low muscle mass) and age-matched healthy controls. Compared with DEXA, effect sizes for RF_csa_ and Q_t_ were larger, which may suggest greater sensitivity to the intervention with ultrasound. Both RF_csa_ and Q_t_ demonstrated good inter-occasion and inter-operator reproducibility, and correlated well at baseline with measurements of muscle size obtained by DEXA. However the changes in measures of quadriceps muscle mass detected by ultrasound and DEXA following training were poorly correlated, suggesting that these measurement methods are not interchangeable when assessing the response to an intervention.

To our knowledge, this is the first study to assess ultrasound for quantifying the effects of RT on indices of quadriceps mass in COPD. Two measures of quadriceps muscle mass-rectus femoris cross sectional area (RF_csa_) and quadriceps thickness (Q_t_), were determined in this study. Both these ultrasound-derived measures have previously been used as surrogate markers of quadriceps size in healthy older populations [10]. Studies have demonstrated the sensitivity of serial ultrasound measurements to detect changes in quadriceps mass in critically ill patients [9] and in healthy populations following exercise interventions [13,14].

In the present study, we observed a significant increase in rectus femoris cross-sectional area and quadriceps thickness after 8 weeks of knee extensor RT in patients with COPD and age-matched healthy controls. Thigh muscle mass measured by DEXA was also increased after training in both groups. The magnitude of post-training changes in muscle mass determined by ultrasound and DEXA in this study is comparable to training data from other patient populations. Jones at al. showed that 6 weeks of lower limb isokinetic RT in healthy young volunteers resulted in approximately a 4.5% increase in DEXA-measured thigh lean mass [16]. In older men with prostate cancer, 20 weeks of progressive RT lead to a 15.7% increase in quadriceps thickness measured by ultrasound [21]. Similarly a 20% increase in rectus femoris cross-sectional area was observed in post menopausal women after 6 months of lower limb RT [12]. Our study provides additional data to support the use of ultrasound as a bedside imaging modality for serial measurements of the quadriceps during RT in COPD patients.

We observed good inter-operator and inter-occasion reproducibility of ultrasound measurements in this study. None of the operators had any previous ultrasound experience. However, after a familiarisation period of 10 to 14 days, all the operators became competent at performing the scans independently. Non clinicians can therefore be easily trained to perform the leg ultrasound scans. In addition, the reproducibility and sensitivity of both ultrasound indices of muscle mass – RF_csa_ and Q_t_, were noted to be similar. Although RF_csa_ may correlate better with muscle strength [10] and is less prone to operator-dependent errors, it may be difficult to measure in certain individuals, such as those with excess or very little fat in the thighs [11]. The intermuscular septae are not clearly visualised in these patients, and measuring Q_t_ may be more appropriate in this situation.

When compared with ultrasound, the post-training changes in muscle mass measured by DEXA were more closely related to changes in QMVC, although this was not statistically significant. A number of factors may account for this lack of correlation between changes in QMVC and changes in muscle mass measured by both ultrasound and DEXA: (i) The rectus femoris constitutes only about 10% of the total cross-sectional area of the quadriceps muscle [22], while measurement of muscle thickness at the mid-thigh region excludes two major muscles belonging to the quadriceps group – the vastus lateralis and vastus medialis. On the other hand, the inability of DEXA measurements to differentiate between flexor and extensor muscles within a limb is a limitation. Therefore, indices of quadriceps mass determined by DEXA and ultrasound do not measure the mass of the whole knee extensor muscle. (ii) The differential response to training between two and three dimensional measurements may be relevant. The rectus femoris has a comparatively large surface area to volume ratio as it is a small muscle. Hence small changes in mass might cause larger changes in measured cross-sectional area of the rectus femoris when compared to DEXA, which measures a larger muscle. (iii) There is data to suggest that small changes in skeletal mass following RT may be undetected by DEXA [23], which is not the case with ultrasound [12,21].

The recognition of the functional and prognostic importance of reduced skeletal muscle mass has intensified interest in developing interventions to address this issue. RT has been shown to be effective in this respect but there is continuing interest in pharmacological and nutritional therapies aimed at achieving this either alone or in conjunction with training. Developing intelligence about the reproducibility and sensitivity of measurements of total and regional muscle mass is a key part of this endeavour. Muscle mass may fall rapidly during a period of acute illness such as an acute exacerbation of COPD [24], and tools to record these changes are needed. Ultrasound is a sensitive and reproducible test that could be used for repeat testing in clinical trials or in situations such as acute illness. It has a number of advantages in these settings including its potential use at the bedside and its lack of ionising radiation, which makes it a useful tool for performing serial measurements. The technique is relatively easy for non radiologists to learn, as its increasing use in other situations such as the insertion of intercostal chest drains for pleural disease illustrates.

We recognise that ultrasound has limitations when compared to other methods such as DEXA. Measurement errors can occur with the use of this technique as it is more operator-dependent than other imaging techniques. This can be minimised by avoiding excessive tissue compression during scanning and ensuring that the probe is always placed perpendicular to the long axis of the limb being measured. As previously discussed, visualisation of intermuscular septae may be difficult in severely obese subjects [11] and in those with tissue depletion, hence accurate measurements of rectus femoris cross-sectional may not be obtainable [25]. DEXA measurements on the other hand are not operator dependent, and it gives information on whole-body and regional limb body composition, including bone mineral and lean tissue mass. DEXA may therefore perform better than ultrasound as a method for screening and identifying nutritional depletion [26], whereas ultrasound may be best used as an assessment tool to measure the response to intervention. The inclusion of an untrained control group would have allowed a more robust assessment of the impact of RT on muscle mass and provided information on the longer term biological variability of the measurement. However, our objective was the investigation of the sensitivity of ultrasound in comparison with other measurement methods rather than the impact of training per se.

In conclusion, indices of quadriceps mass measured by portable ultrasound are reproducible and sensitive to change in response to knee extensor RT in COPD. Our data suggest the potential for ultrasound to be used as a field measurement of lower limb muscle mass in this population, whilst also highlighting limitations of the technique. The differences in the response to the intervention between ultrasound and DEXA suggest these measurements reflect different anatomical characteristics of the lower limb muscles and are not interchangeable.

## Abbreviations

RT: Resistance training; DEXA: Dual energy x-ray absorptiometry; QMVC: Quadriceps maximum voluntary contraction; RF_csa_: Rectus femoris cross-sectional area; Q_t_: Quadriceps thickness; T_dexa_: Thigh lean mass.

## Competing interests

The authors declare that they have no competing interests.

## Authors' contributions

Drs MKM and MCS contributed to the study concept and design. Dr MKM, Dr LH and Miss SH were responsible for data collection. Drs MKM, LH, and MCS contributed to the analysis and interpretation of the data. Dr MKM was responsible for drafting of the article. Drs SJS, MDM, and MCS contributed to the critical revision of the article for important intellectual content. Drs MKM and MCS take responsibility for the integrity of this work as a whole. All authors read, commented on, and contributed to the submitted manuscript. All authors read and approved the final manuscript.

## Funding

This research was supported by a project grant from the UK Medical Research Council (G0501985).
